# Transactions of the American Medical Association, Volume 5th, 1852

**Published:** 1853-03

**Authors:** 


### EDITORIAL.

*Transactions of the American Medical Association, Volume 5th,* 1852.

THE above is the title of a volume containing the proceedings, reports, and papers of the Association at its fifth annual meeting, held at Richmond, Va., May, 1852, embracing no less than 939 large octavo pages.

Glancing over this ponderous volume, we thought it might not be unprofitable to give the readers of the *North-Western Medical and Surgical Journal* some idea of its contents, with the hope of awakening more attention to its value, and more interest in the permanency and success of the Association itself. To do this will require more space than can be afforded in one number of the Journal, and more time than is now at my command. Hence, with due permission, I will notice the several parts of the volume in successive numbers of the Journal, beginning with the "Minutes of the fifth annual meeting." From these we learn that the meeting was composed of 275 members, representing the societies and institutions of 32 states, the district of Columbia, the United States navy, and the American Medical Society of Paris. Of the 275 members, 39 are registered as representatives of medical colleges, embracing 26 different institutions; 7 represented the same number of hospitals; 2 belonged to the Baltimore Infirmary; 1 to the United States Navy; and the remaining 226 were representatives of city, county, district, and state societies.

The only thing worthy of notice in the first day's proceedings, besides the election of officers, was the report of the committee on prize essays, from which we learn that sixteen essays were presented to the committee for examination. Only one prize was awarded, which was received by Dr. Austin Flint of Buffalo, for the essay noticed in the preceding number of this Journal. On the morning of the second day, Dr. Simons of S. C., very properly called the attention of the Association to the great and unnecessary destruction of human life, which results from the crowding of emigrant ships, and proposed the following resolutions, which were adopted, viz:

"*Resolved*, that the American Medical Association do memorialize Congress to require all vessels carrying steerage passengers on the sea, to have a surgeon on board;

*Resolved*, that a committee of the association be appointed to draw up a memorial to Congress, making such suggestions as it may deem fit as regards the importance of this measure, and also the importance of giving to each steerage passenger a certain amount of space between decks."

This is a very important subject; for the frequent generation of severe and fatal diseases on board of crowded emigrant ships, and their transference from thence into all our sea-port towns and commercial cities, is a much more serious cause of disease than is generally supposed; and it is the duty of physicians throughout the whole country to call the attention of members of Congress to this subject.

Only two other items particularly attracted my attention in the *minutes*. The first was the very small amount of time devoted to the reading and discussion of reports and papers on scientific subjects. Nearly all the matter of this kind was postponed until the last day of the session, and was then disposed of under the following motion, viz.:

"The reports of the committee on scientific subjects being called for, Dr. Horner, of Pa., moved that they be *read* by *their titles*, and referred to the committee of publication; *which motion was adopted*."

In this summary manner they disposed of the matter which fills at least six hundred of the 939 pages that constitute the volume of transactions; and to perpetuate this *time*-saving process, they also adopted the following resolution, viz.:

"*Resolved*, That at the future meetings of this Association, all reports of committees and all contributions on scientific subjects, occupying more than ten pages of quarto post manuscript, be accompanied each by an abstract or synopsis embracing the principal points of such report or paper, which abstract or synopsis shall be read before the association."

With all due deference to the wisdom of the Association, I most emphatically express my disbelief in the correctness of this policy. To appoint at each meeting a list of twenty or thirty special committees, all instructed to report, and then read those reports by their *titles only*, and refer them for publication, is certainly opening the way for the annual publication of an enormous amount of matter, without knowing whether one-fourth of it is worth the cost of the paper it is printed on. I make this assertion with a full knowledge of the contents of the volume before me, and I am well satisfied that it would render the annual meetings of the Association more interesting, and contribute infinitely more to the advancement of medical science and the elevation of our medical literature, if the Association would appoint a much *smaller number* of committees, and require each paper to be read and discussed in full before being sent to the committee for publication. If this course should be adopted, we might have a *smaller* volume of transactions, but one proportionately more valuable; and if it should induce those who attend the meetings to examine with some care the subjects on which reports and papers were expected, that they might be prepared to discuss creditably such reports, instead of spending a large part of the time of each annual session in wrangling about provisions of the constitution, it would be far more profitable both to themselves and the profession.

The second item that attracted my attention in the minutes, was the large share of time which was consumed in proposing and discussing amendments to the constitution of the Association.

It would appear that much the larger part of the second and third days of the session, was spent on this subject. The same thing has occurred at several previous meetings—indeed there seems to have been two very distinct classes of men in the profession, who have never been satisfied with the present organization of the Association. The first class are emphatically *outsiders.* One or two of their number appeared in the first National Convention, and made an ill-timed effort to break it up and thus arrest the whole movement; but failing in this, they have kept entirely aloof from the subsequent meetings, and apparently contented themselves in publishing, chiefly through an organ established not far from the borders of Canada, the most gross and wanton assaults upon the character of the Association and the motives of its members. This class, having never travelled a mile or spent a *dime* to aid in advancing the interests of the Association they so freely assail, merit only the silent contempt of every honorable member of the profession. The second class have occupied no such "dogin-the-manger" position. They have given their time and money freely in attending the annual meetings, and have shared largely in the honors and responsibilities connected with the institution, but have never been content with the more important provisions of the present constitution. At the very outset they opposed the principle of representation, advocating the idea that all regular members of the profession should be members of the Association and entitled to attend its meetings, whether appointed by any society or public institution, or not. The objections to this were so obvious, however, that the present constitution, with its basis and ratio of representation, was adopted by a large majority. The opposition, notwithstanding, has continued to manifest itself at every meeting since. That the reader may fully understand the merits of this subject, it is necessary to state that the second article of the present constitution provides that *members* of the Association shall consist of delegates appointed by permanently organized medical societies, colleges, and hospitals. Each society is entitled to send *one* delegate for every *ten* of its members; each college *two*; and each hospital, containing a hundred beds, *two*. Finding it impossible to destroy entirely the principle of *representation* in the present organization, the *equality*, or rather want of equality in the present ratio, has become a prominent topic of complaint. It is claimed to be unfair, *anti-republican* I presume, that College faculties, generally numbering not more than seven or eight medical men, should be entitled to *two* members, while *ten* men, not connected with Colleges, are only entitled to *one* member. Hence, after much discussion, and reports from two or three committees, the following provisions were recommended by the last meeting for adoption at the next, as substitutes for articles I. and II. in the present constitution, viz.:

ARTICLE I.—*Title of the Association.*

This institution shall be known and distinguished by the name and title of "The American Medical Association." It shall be composed of all the members of the medical profession of the United States, of good standing, who acknowledge fealty and adhere to the code of ethics adopted by the Association; and its business shall be conducted by their delegates or representatives, who shall be appointed annually in the manner prescribed by this Constitution.

Strike out the whole of Article II., referring to "Members," and insert the following:—

Article II.—*Of Delegates.*

§ 1. The delegates to the meetings of the Association shall collectively represent and have cognizance of the common interests of the medical profession in every part of the United States, and shall hold their appointment from County, State, and regularly chartered Medical Societies; from chartered Medical Colleges, Hospitals, and permanent Voluntary Medical Associations in good standing with the profession. Delegates may also be received from the medical staffs of the United States army and navy.

§ 2. Each delegate shall hold his appointment for one year and until another is appointed to succeed him, and he shall be entitled to participate in all the business affairs of the Association.

§ 3. The county, district, chartered, and voluntary medical societies shall have the privilege of sending to the Association one delegate for every ten of its resident members, and one more for every additional fraction of more than one-half of this number.

§ 4. Every State society shall have the privilege of sending four delegates; and in those States in which county and district societies are not generally organized, in lieu of the privilege of sending four delegates, it shall be entitled to send one delegate for every ten of its regular members, and one more for every additional fraction of more than one-half of this number.

§ 5. No medical society shall have the privilege of representation which does not require of its members an observance of the code of ethics of this Association.

§ 6. The Faculty of every Chartered Medical College, acknowledging its fealty to the code of ethics of this Association, shall have the privilege of sending one delegate to represent it in the Association: *Provided*, That the said faculty shall comprise six professors, and give one course of instruction annually of not less than sixteen weeks on Anatomy, Materia Medica, Theory and Practice of Medicine, Theory and Practice of Surgery, Midwifery, and Chemistry: *And provided also*, that the said faculty requires of its candidates for graduation—1st. That they shall be twenty-one years of age; 2d. That they shall have studied three entire years, two of which must have been with some respectable practitioner; 3d. That they shall have attended two full courses of lectures (not however to be embraced in the same year), and one of which must have been in the institution granting the diploma, and also where students are required to continue their attendance on the lectures to the close of the session; and 4th. That they shall show by examination that they are qualified to practise medicine.

§ 7. The medical faculty of the University of Virginia shall be entitled to representation in the Association, notwithstanding that it has not six professors, and that it does not require three years of study from its pupils, but only so long as the present peculiar system of instruction and examination practised by that institution shall continue in force.

§ 8. All Hospitals, the medical officers of which are in good standing with the profession, and which have accommodation for one hundred patients, shall be entitled to send one delegate to the Association.

§ 9. Delegates representing the Medical Staffs of the United States army and navy shall be appointed by the Chiefs of the Army and Navy Medical Bureaux. The number of delegates so appointed shall be four from the army medical officers and an equal number from the navy medical officers.

§ 10. No delegate shall be registered on the books of the Association as representing more than one constituency.

§ 11. Every delegate elect, prior to the permanent organization of the annual meeting, and before voting on any question after the meeting has been organized, shall sign the Constitution and inscribe his name and address in full, with the title of the institution which he represents.

Every reader who makes the comparison, will notice three prominent differences between these articles and the two for which they are destined as substitutes. The first consists in the proposition to make "all the members of the medical profession of the United States, of good standing, who acknowledge fealty and adhere to the code of ethics adopted by the Association," in fact, members of the same. The idea contained in this proposition has been advocated by some eminent members of the Association ever since its organization. And yet, how it is to accomplish the least possible good, it is not easy to perceive. On the contrary, it opens a wide door for practising the most annoying abuses. It is well known that no class of men are more fond of adopting the *titles* which might be supposed to indicate an *honorable* position in our profession, than the whole motley group of quacks whom we repudiate, and a few of our own members who feel the need of some dignified title to give them that consideration with the public, which their own want of mental industry and acquirements fail to obtain for them. Let the proposition be adopted by the Association, and what is to hinder any man who chooses to style himself "a member of the medical profession," from assuming the title of "Member of the American Medical Association?"

True, the proposition requires that he be "of good standing," and that he "acknowledge fealty to the code of ethics;" but who is to be the judge of his standing? and what provision is made or proposed for ascertaining it? This is not the only fault, however. There is no more laudable or powerfully operating stimulus to human exertion, of a temporal nature, than the honest desire to gain a fair and honorable position among our fellows; and the opportunities which medical societies afford for the gratification of this desire, constitute by no means the least important of their benefits. To become members in free and friendly communion of a local society; to gain a position in that society which will enable us to represent it in the State society; and to go still further, and acquire the privilege of mingling, as a representative, in the great national organization, and there forming the acquaintance and listening to the discussions of the most eminent men in our profession, are so many powerful incentives to a just and honorable emulation throughout the ranks of the profession in every State of our broad confederacy.

But let the National Association adopt a constitutional provision, which will enable every brazen-faced pretender in medicine to arrogate to himself the title of member of the "American Medical Association," even without the necessity of becoming first a member of a local society where he lives, and one of its most efficient operative influences for good is at once greatly diminished, if not destroyed. As the constitution of the Association now stands, with the only avenue to membership through local and State societies, colleges, and hospitals, it holds out the strongest possible inducements for the formation of city, county, and State societies; and, with its plan of holding annual meetings in different and distant sections of the Union, it has already done a great and beneficial work in extending the organization of the profession in a large number of States, in which scarcely any organization existed before. Why then abandon, or at least diminish, these great and manifest advantages, for the sake of conferring a mere nominal membership on the whole profession, which, from its very commonness, will be least esteemed by those who ought to be most interested in the welfare of the Association?

The second point of difference between amendments proposed an the present provisions of the constitution, is in the ratio of representation from colleges and hospitals. It is proposed to limit each of these institutions to *one* delegate instead of *two*, as at present. This proposition seems to be a kind of *compromise* between the friends of the present constitution and those who desire to abolish the provision for delegates from such institutions altogether. At the organization of the Association, it was very generally thought to be an object of great importance, to secure the active and hearty co-operation of the Medical Colleges. The free intermingling annually of delegates from those institutions with others from the great mass of the profession, by which each would become more familiar with the wishes, embarrassments, and advantages of the other, was thought to be the most desirable and certain method of securing this important end. The framers of the constitution, having much more reference to the accomplishment of a great object than to the attainment of a nice numerical equality in the ratio of representation, provided for the admission of two delegates from each college and hospital. Has anything occurred in the practical working of the provision that calls for a change? In casting a hasty glance over the list of delegates in attendance at each annual meeting of the Association since its formation, I find the ratio of representation to be substantially as follows, viz.:

| | 1847 | 1848 | 1849 | 1850 | 1851 | 1852 |
|---|---|---|---|---|---|---|
| Members enrolled as Delegates from Societies.... | 178 | 208 | 375 | 208 | 182 | 226 |
| " " " " Med. Col..... | 59 | 45 | 51 | 34 | 30 | 39 |
| " " " " Hospitals.... | 2 | 24 | 20 | 13 | 10 | 9 |

Total Average from Medical Societies during six years.....................230
" " " " Colleges " " " ....... ............. 42
" " " Hospitals and Infirmaries during six years................ 13

Certainly these figures furnish no evidence that medical schools have hitherto been too largely represented, the actual average number in attendance having scarcely exeeeded *one* for each school in the Union. Hence, if they are allowed any representation, the benefits to result from reducing their ratio are not very apparent.

The third important difference between the amendments proposed and the present constitution, consists in the conditions imposed on the colleges to entitle them to send delegates to the Association. These conditions are essentially as follows, viz.: The college faculty must embrace at least six professors; the session to occur but once a year, and continue full sixteen weeks; the candidates for graduation must be required to be 21 years of age, to have studied *three* entire years, to have attended two full courses of lectures, and to sustain a satisfactory examination.

These conditions are all right enough in themselves, but entirely out of place in the *constitution* of a great National Association. They constitute unnecessary details that almost inevitably involve the Association in the most glaring inconsistencies. Indeed, the very next section of this same proposed amendment, affords a striking instance of such inconsistencies. In this, to avoid the awkward necessity of excluding the University of Virginia, the association is constrained by the terms of the amendment to acknowledge, that, under some circumstances, neither *six* professors, nor *three* years study, nor *two* courses of lectures are necessary; and that, too, after their annually reiterated recommendation of *longer* college terms, *longer* periods of study, a *greater* number of courses of lectures, and *seven* professors. It seems to me that all this talk and hair-splitting nicety about constitutional provisions, for an Association organized for the purpose of elevating and advancing the condition of one of the most learned and beneficent professions that exist in human society, involves an unnecessary waste of most precious time; and is far better calculated to engender jealousies and divisions, than to foster that spirit of liberal friendship and noble zeal for the advancement of our science, which should extend their genial influences to the remotest corners of our country. But if eminent members of the Association persist in having these *detailed conditions* in reference to medical schools incorporated into the constitution, I should certainly recommend very earnestly one additional condition to be exacted of all medical societies before they should be entitled to a representation. At the first meeting of the Association held in Philadelphia, 1847, the following resolution was adopted by an almost unanimous vote, viz.: "Resolved, That this Convention earnestly recommends to members of the profession throughout the United States, to satisfy themselves, *before* receiving young men into their offices as students, that they are of good moral character, and *that they*

*have acquired a good English education, a knowledge of Natural Philosophy, and the Elements of Mathematical Sciences, including Geometry and Algebra ; and such an acquaintance, at least, with the Latin and Greek languages, as will enable them to read and write prescriptions.*"

If we are to have detailed *constitutional* conditions exacted of schools and hospitals, let us also require every Society to adopt efficient regulations for carrying the above resolutions into full practical effect, by all its members. Such a step, once accomplished, would certainly do more to elevate the character and extend the usefulness of the profession, than any other measure that human ingenuity can devise. Let no one imagine, that in making the foregoing strictures on the doings of the last meeting of the Association, I am inclined to find unnecessary fault, or to interpose the slightest obstacle to the prosperity and harmony of that institution. On the contrary they are called forth by the warmest solicitude for its permanent prosperity and usefulness. It has, during the six brief years of its existence, done much, very much, to create a healthier public sentiment in the profession, to increase the intercourse and friendly feelings among its members, to extend its local organizations, and to excite the feeling of just emulation in the pursuit of science.

I ardently desire to see it continue to diffuse abroad the same healthful and ennobling influences. While I admit that the present constitutional ratio of representation is *theoretically* unequal, yet it certainly has not, thus far, developed in practice any important abuses. And hence, instead of risking the harmony of diverse interests and feelings by constant attempts at more nice constitutional adjustments, every effort should be made to cultivate a broader liberality, a freer communion of those representing all legitimate interests, and a more patient, diffusive, and energetic spirit of scientific research. And I am fully convinced that these important objects would be more speedily and certainly accomplished, if the Association would appoint fewer committees, exact more prompt and carefully digested reports, and induce all its members to come up to the annual meetings prepared in the true spirit of candor and liberality, to examine every report and paper presented, for the purpose of adding whatever their own experience and observation had furnished in relation thereto. Such a course, pursued in such a spirit, would not only carry the Association onward through a long career of prosperity and usefulness, but would give to all its general recommendations, in relation to medical education and literature, greatly increased effect. But enough. N. S. D.

